# Prevalence of Aflatoxin B1 and Other Quality Indicators in Artisanal Peanut Oil from Small-Scale Workshops in Nanning, China

**DOI:** 10.3390/foods15193493

**Published:** 2026-09-29

**Authors:** Zihao Qi, Fan Qiao, Yu Xie, Huishuai Li, Liumin Wei, Huiyan Qin, Haibo Wang, Zhenning Li, Liming Huang, Feiyu Zhao, Binbin Yang, Dongjie Peng, Shaojun Li

**Affiliations:** 1 Department of Toxicology, School of Public Health, Guangxi Medical University, Nanning 530021, China; 2 Guangxi Colleges and Universities Key Laboratory of Prevention and Control of Highly Prevalent Diseases, Guangxi Medical University, Nanning 530021, China; 3 Guangxi Key Laboratory of Environment and Health Research, Guangxi Medical University, Nanning 530021, China; 4 Guangxi Youqingmiyi Technology Co., Ltd., Liuzhou 545104, China; 5 Guangxi Zhuang Autonomous Region Center for Disease Control and Prevention, Nanning 530028, China; 6 Guangxi-ASEAN Food Inspection Center, Nanning 530029, China; 7 Department of Maternal & Child and Adolescent Health, School of Public Health, Guangxi Medical University, Nanning 530021, China

**Keywords:** artisanal peanut oil, aflatoxin B1, food safety, quality profiles, dietary exposure, risk assessment

## Abstract

Objective: To characterize aflatoxin B1 (AFB1) contamination, oil-quality variation, workshop hygiene, and modeled dietary risk associated with artisanal peanut oil produced by small-scale workshops in Nanning, China. Methods: A cross-sectional study was conducted in 144 workshops across all 12 county-level administrative areas of Nanning, with one peanut-oil sample collected from each workshop. On-site hygiene practices were assessed using a structured 13-item instrument. AFB1 concentration, acid value, and peroxide value were measured using national standard methods. Regional differences were evaluated using non-parametric tests, and K-means clustering was applied to characterize multivariate quality profiles. Dietary exposure was assessed using estimated daily intake (EDI), margin of exposure (MOE), and modeled AFB1-attributable hepatocellular carcinoma (HCC) risk. Results: AFB1 was detected in 88.2% of samples, while 3.5% exceeded the Chinese maximum level of 20 μg/kg. Samples from counties had significantly higher AFB1 concentrations, acid values, and peroxide values than those from municipal districts (all *p* < 0.001). Compliance was relatively high for basic hygiene and spatial separation, whereas routine contaminant testing was reported by only 18% of workshops and aflatoxin-degradation equipment was available in 35%. The primary clustering analysis identified overall-low (81.2%), AFB1-dominant (9.7%), and rancidity-dominant (9.0%) profiles, whereas sensitivity analysis after log_10_ transformation favored a broader two-cluster structure. Estimated AFB1 intake was higher under the rural than the urban scenario (2.6436 vs. 0.6890 ng/kg bw/day), with corresponding MOE values of 151.31 and 580.55. Modeled HCC risk was also higher under the rural scenario and under the hepatitis B surface antigen-positive scenario. Conclusions: Artisanal peanut oil from small-scale workshops in Nanning showed frequent AFB1 detection, marked regional variation in multiple quality indicators, and limited implementation of contaminant-specific control practices. These findings support closer attention to routine AFB1 monitoring, storage management, and broader quality control, particularly in county settings.

## 1. Introduction

Aflatoxins are toxic secondary metabolites produced mainly by aflatoxigenic Aspergillus species [1]. Among the aflatoxins occurring in foods, aflatoxin B1 (AFB1) is of greatest toxicological concern because of its potent genotoxic and carcinogenic effects [1,2]. Peanuts are particularly susceptible to aflatoxin contamination both before harvest and during post-harvest handling. Drought and high-temperature stress during cultivation can increase the risk of pre-harvest contamination, whereas inadequate drying and storage under favorable temperature and moisture conditions can promote fungal growth and further aflatoxin accumulation after harvest [3,4,5,6]. Nanning, the capital of Guangxi in southern China, has a subtropical monsoon climate characterized by warm temperatures and abundant rainfall [7]. Under such conditions, inadequate post-harvest drying or storage may further increase the susceptibility of peanuts to fungal growth and aflatoxin contamination [8].

Although AFB1 has limited solubility in the lipid phase, disruption of contaminated peanut kernels during extraction may result in its carryover into crude oil, with the extent of transfer varying according to the initial contamination level, kernel pretreatment, and extraction conditions [9,10]. Previous studies have reported substantial geographic variation in aflatoxin contamination of peanuts and peanut oil across China [11]. Recent surveillance studies in China have likewise identified edible oils, including peanut oil, as relevant matrices for aflatoxin occurrence and dietary exposure [12,13,14]. In Guangdong Province, for example, AFB1 was detected in 79.7% of 143 unpackaged peanut oil samples, with concentrations ranging from 0.02 to 174.13 μg/kg; some samples exceeded the maximum level of 20 μg/kg specified for AFB1 in peanut oil under GB 2761-2017 [15,16]. These findings indicate that detectable AFB1 contamination remains common in unpackaged peanut oil in southern China, although the magnitude of contamination varies considerably among regions and production settings [11,15].

Small-scale peanut-oil workshops represent an important setting for food-safety surveillance because raw-material handling, storage, processing, hygiene management, and routine quality-control practices may differ among producers [17]. In this study, artisanal peanut oil refers to peanut oil produced on-site by small-scale family-operated workshops using their own processing equipment. A recent study in southern China reported reductions in AFB1 contamination following the implementation of management regulations for small food workshops, highlighting the relevance of production-level oversight [17]. Previous investigations have primarily focused on AFB1 occurrence, regulatory exceedance, or dietary exposure in peanut oil [11,15,17]. However, evidence integrating AFB1 occurrence and concentration with other oil-quality indicators, regional variation, and workshop hygiene practices remains limited. Evaluating these dimensions together may provide a more complete characterization of the heterogeneity in the quality and safety of peanut oil produced by small-scale workshops.

Primary liver cancer remains a major global health burden, with particularly high incidence and mortality rates in Eastern Asia [18]. Dietary exposure to AFB1 is also of public-health concern because AFB1 is a genotoxic carcinogen associated with hepatocellular carcinoma (HCC), with substantially greater carcinogenic potency in populations with chronic hepatitis B virus infection [1,19,20]. This cross-sectional study therefore aimed to characterize the quality and safety of artisanal peanut oil produced by small-scale workshops across Nanning. Specifically, we assessed AFB1 occurrence and concentration together with acid and peroxide values, documented workshop hygiene and AFB1-control practices, examined regional differences and multivariate quality profiles, and estimated dietary AFB1 exposure and modeled HCC risk under urban and rural exposure scenarios.

## 2. Methods

### 2.1. Study Design and Sample Collection

This cross-sectional study was conducted in Nanning, Guangxi, China, covering all 12 county-level administrative divisions of the city, including seven municipal districts and five counties. A stratified random sampling design with equal allocation was used, with each administrative division treated as a stratum. Twelve eligible peanut-oil workshops were randomly selected from each stratum, yielding 144 workshops in total, including 84 from municipal districts and 60 from counties. One peanut-oil sample was collected from each workshop.

As a precision check, a previously reported AFB1 prevalence of 79.7% in unpackaged peanut oil from southern China was used as the reference estimate [15]. With 144 independent samples, the corresponding 95% confidence interval half-width was approximately 6.6 percentage points.

Eligible workshops were family-operated establishments that produced peanut oil on-site using their own processing equipment and had been in continuous operation for at least 3 months. Eligibility was verified during field visits based on raw-material procurement records, production equipment, and records documenting the source of the oil produced. Retail-only outlets, temporary stalls, and establishments for which the production source could not be verified were excluded. For the purposes of this study, artisanal peanut oil was defined as peanut oil produced on-site by these eligible small-scale workshops. Only freshly pressed oil produced within 72 h before sampling was included.

Approximately 500 mL of peanut oil was collected from each workshop in a 500-mL amber glass container with a screw-cap closure. Samples were protected from light and transported under refrigerated conditions at approximately 4 °C, stored at 4 °C upon arrival at the laboratory, and analyzed within 48 h of collection.

### 2.2. On-Site Hygiene Assessment

A structured 13-item assessment was developed with reference to the National Food Safety Standard—Hygienic Specifications for the Production of Edible Vegetable Oils and Their Products (GB 8955-2016) [21] and the National Food Safety Standard—Vegetable Oils (GB 2716-2018) [22]. The assessment covered three domains: production environment hygiene, personnel and raw-material management, and aflatoxin prevention and control (Supplementary Table S1).

Information was collected through a combination of direct on-site observation and structured interviews with workshop personnel. Conditions that could be directly assessed, including the production environment, storage conditions, equipment cleanliness, and spatial separation of production areas, were recorded by trained investigators during site visits. Management practices that could not be reliably determined by observation, including routine contaminant testing and AFB1-control measures, were documented through structured interviews. Each assessment item was classified as compliant or non-compliant, and compliance was expressed as the number and percentage of workshops meeting the corresponding criterion. Completed assessment records were independently checked by two researchers for completeness and consistency.

### 2.3. Laboratory Analysis

All laboratory analyses were performed in a China Metrology Accreditation (CMA)-accredited laboratory in accordance with the relevant Chinese national food-safety standards.

AFB1 was quantified using immunoaffinity-column purification followed by high-performance liquid chromatography with fluorescence detection (HPLC-FLD), according to GB 5009.22-2016 (Determination of Aflatoxins in Foods) [23]. The limit of detection (LOD) was 0.05 μg/kg.

Acid value was determined by cold-solvent automatic potentiometric titration according to GB 5009.229-2016 (Determination of Acid Value in Foods), and peroxide value was measured by titration according to GB 5009.227-2016 (Determination of Peroxide Value in Foods) [24,25]. Duplicate analyses were included in each analytical batch for quality control, together with spike-recovery testing. Recovery rates ranged from 85% to 115%.

### 2.4. Statistical Analysis

All statistical analyses were performed using R software (version 4.2.1) [26]. AFB1 prevalence was defined as the proportion of samples with concentrations at or above the LOD (0.05 μg/kg), whereas samples below the LOD were classified as non-detections. The proportion of samples exceeding the Chinese maximum level of 20 μg/kg was calculated separately. Results from the on-site hygiene assessment were summarized as frequencies and percentages.

Normality of AFB1 concentration, acid value, and peroxide value within the municipal-district and county groups was assessed using the Shapiro–Wilk test. Because all three indicators showed significant departures from normality, differences between municipal districts and counties were evaluated using the Mann–Whitney U test. Regional distributions were summarized using medians and interquartile ranges, with means and standard deviations additionally reported where appropriate for descriptive characterization. All statistical tests were two-sided, and *p* < 0.05 was considered statistically significant.

For multivariate quality-profile analysis, AFB1 concentration, acid value, and peroxide value were standardized as Z-scores before K-means clustering. All 144 samples had complete measurements and were included in the analysis. Extreme observations were checked against the original analytical records, and no observations were excluded solely on the basis of statistical extremity. Candidate solutions from k = 2 to k = 6 were evaluated using the elbow method, silhouette coefficient, and Gap statistic [27,28]. The primary cluster solution was selected on the basis of the overall clustering evidence, parsimony, and interpretability of the resulting profiles. Cluster characteristics were described using standardized cluster centroids together with the distributions of the original measurements.

A sensitivity analysis was performed using log_10_-transformed data. For AFB1, non-detections were assigned one-half of the LOD (0.025 μg/kg) before transformation; acid and peroxide values were directly log_10_-transformed because all observations were positive. The transformed variables were subsequently standardized as Z-scores, and K-means clustering was repeated across k = 2–6. For direct comparison with the primary three-cluster solution, a three-cluster model was also fitted to the transformed data. Agreement between the primary and transformed three-cluster solutions was assessed using the adjusted Rand index and the proportion of samples retaining the same profile assignment [29].

Data visualization was performed using the ggplot2 package [30].

### 2.5. Dietary Exposure and Health Risk Assessment

Dietary exposure to AFB1 was estimated using the estimated daily intake (EDI):

$$\mathrm{EDI}=\frac{C\times \mathrm{IR}}{\mathrm{BW}}$$

where C is the mean AFB1 concentration in peanut oil (μg/kg), IR is the daily edible-oil intake (g/day), and BW is the average body weight (kg). With these units, EDI was expressed as ng/kg bw/day. Dietary intake and body-weight data were obtained from the Report on Nutrition and Health Status of Residents in Guangxi (2010–2012) [31]. For adults aged 18–60 years, the values used were 27.03 g/day and 59.27 kg for urban residents and 25.18 g/day and 54.47 kg for rural residents, respectively.

The margin of exposure (MOE) was calculated as:

$$\mathrm{MOE}=\frac{\mathrm{BMD}L_{10}}{\mathrm{EDI}}$$

where the benchmark dose lower confidence limit for a 10% response (BMDL10) for AFB1 was set at 0.4 μg/kg bw/day (400 ng/kg bw/day) [1].

AFB1-attributable hepatocellular carcinoma (HCC) risk was estimated by multiplying EDI by the corresponding carcinogenic potency factor. Separate model-based scenarios were evaluated for hepatitis B surface antigen (HBsAg)-negative and HBsAg-positive populations using potency factors of 0.01 and 0.30 cases per 100,000 person-years per ng/kg bw/day, respectively [32].

For the urban exposure scenario, the mean AFB1 concentration measured in samples collected from the seven municipal districts was combined with the corresponding urban dietary-intake and body-weight parameters. For the rural exposure scenario, the mean AFB1 concentration measured in samples collected from the five counties was combined with the corresponding rural parameters.

## 3. Results

### 3.1. Hygiene Conditions and AFB1 Control Practices in Small-Scale Workshops

Compliance with the evaluated hygiene and food-safety practices varied across the 144 small-scale workshops (Table 1). Within the production environment hygiene domain, relatively high compliance was observed for adequate ventilation (87%), absence of garbage accumulation (78%), and appropriate temperature and moisture conditions during storage (78%). Lower compliance was observed for clean floors and walls (61%) and equipment free of oil residues (60%).

Within the personnel and raw-material management domain, 84% of workshops maintained separation between poultry or pet areas and production areas, and the same proportion maintained separation between production and living areas. Raw materials and equipment were protected from potential sources of contamination in 88% of workshops. Required production qualification information was prominently displayed in 68% of workshops, whereas 53% of production personnel wore clean work clothing.

Aflatoxin-specific control practices were less commonly implemented. Routine contaminant testing was reported by 18% of workshops, while aflatoxin-degradation equipment was available in 35%; 62% reported an intention to acquire such equipment. Overall, compliance was generally higher for basic environmental hygiene and spatial separation measures than for routine contaminant monitoring and aflatoxin-specific control practices.

### 3.2. AFB1 Occurrence and Regional Variation in Peanut Oil Quality Indicators

AFB1 was detected in 127 of the 144 peanut oil samples (88.2%). Despite the high detection frequency, only five samples (3.5%) exceeded the maximum level of 20 μg/kg. AFB1 concentrations ranged from below the LOD to 29.605 μg/kg.

**Figure 1:**
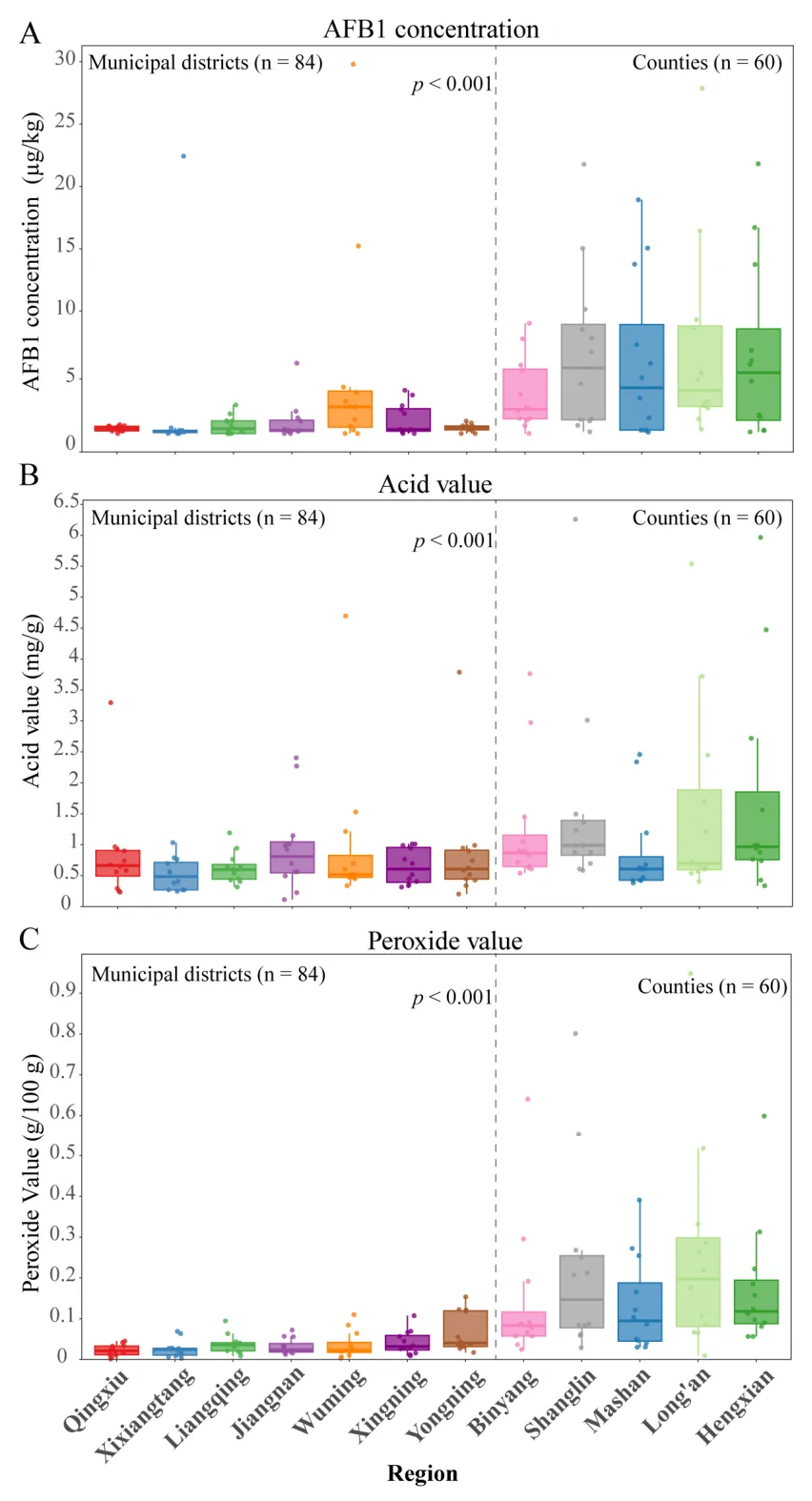
Regional distributions of peanut oil quality indicators in Nanning. (**A**) AFB1 concentration; (**B**) acid value; and (**C**) peroxide value. The dashed vertical line separates municipal districts (*n* = 84) from counties (*n* = 60). Differences between the two groups were assessed using the Mann–Whitney U test (*p* < 0.001 for all indicators).

Clear regional differences were observed across all three quality indicators. Samples from counties had a substantially higher median AFB1 concentration than those from municipal districts (3.554 vs. 0.332 μg/kg; *p* < 0.001; Figure 1). Similar differences were observed for acid value (0.875 vs. 0.583 mg/g; *p* < 0.001) and peroxide value (0.1004 vs. 0.0276 g/100 g; *p* < 0.001). The corresponding Mann–Whitney U statistics were 963, 1567, and 566 for AFB1 concentration, acid value, and peroxide value, respectively (Supplementary Table S2).

Overall, samples collected from counties consistently showed higher levels of AFB1 and both deterioration-related quality indicators than those collected from municipal districts, indicating a clear regional contrast in peanut oil quality.

### 3.3. Multivariate Quality Profiles of Peanut Oil

**Figure 2:**
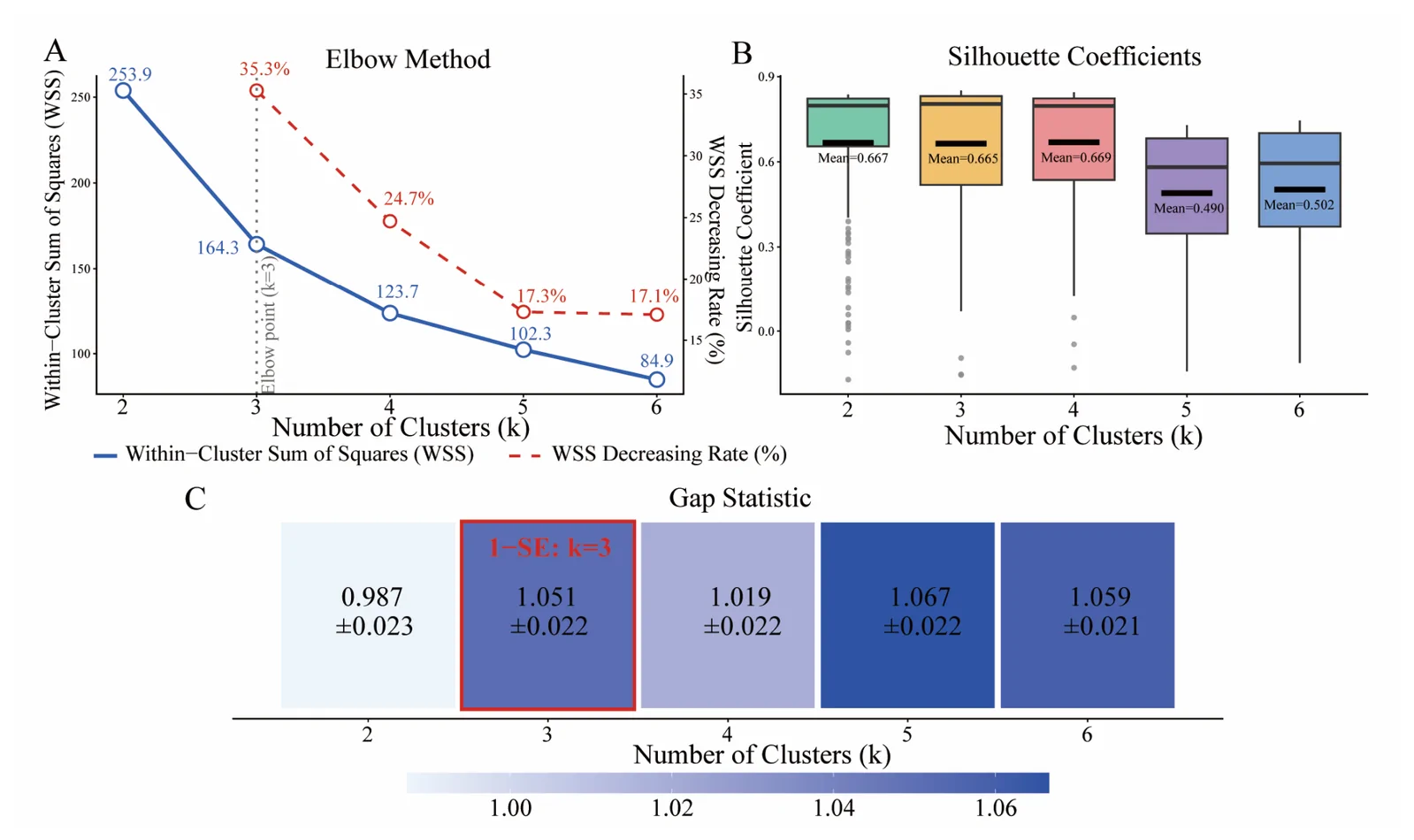
Evaluation of the number of clusters for K-means analysis. (**A**) Elbow method based on the within-cluster sum of squares (WSS); (**B**) silhouette coefficients for k = 2–6; and (**C**) gap statistic. The elbow method and the one-standard-error (1-SE) criterion for the gap statistic supported k = 3.

K-means clustering of standardized AFB1 concentration, acid value, and peroxide value was evaluated across solutions from k = 2 to k = 6. The elbow method and Gap statistic favored k = 3 (Figure 2). Although the mean silhouette coefficient was marginally higher for k = 4 than for k = 3 (0.6688 vs. 0.6645), the difference was minimal (Δ = 0.0043). Given this small difference and the greater parsimony of the three-cluster solution, k = 3 was retained for primary descriptive characterization.

**Figure 3:**
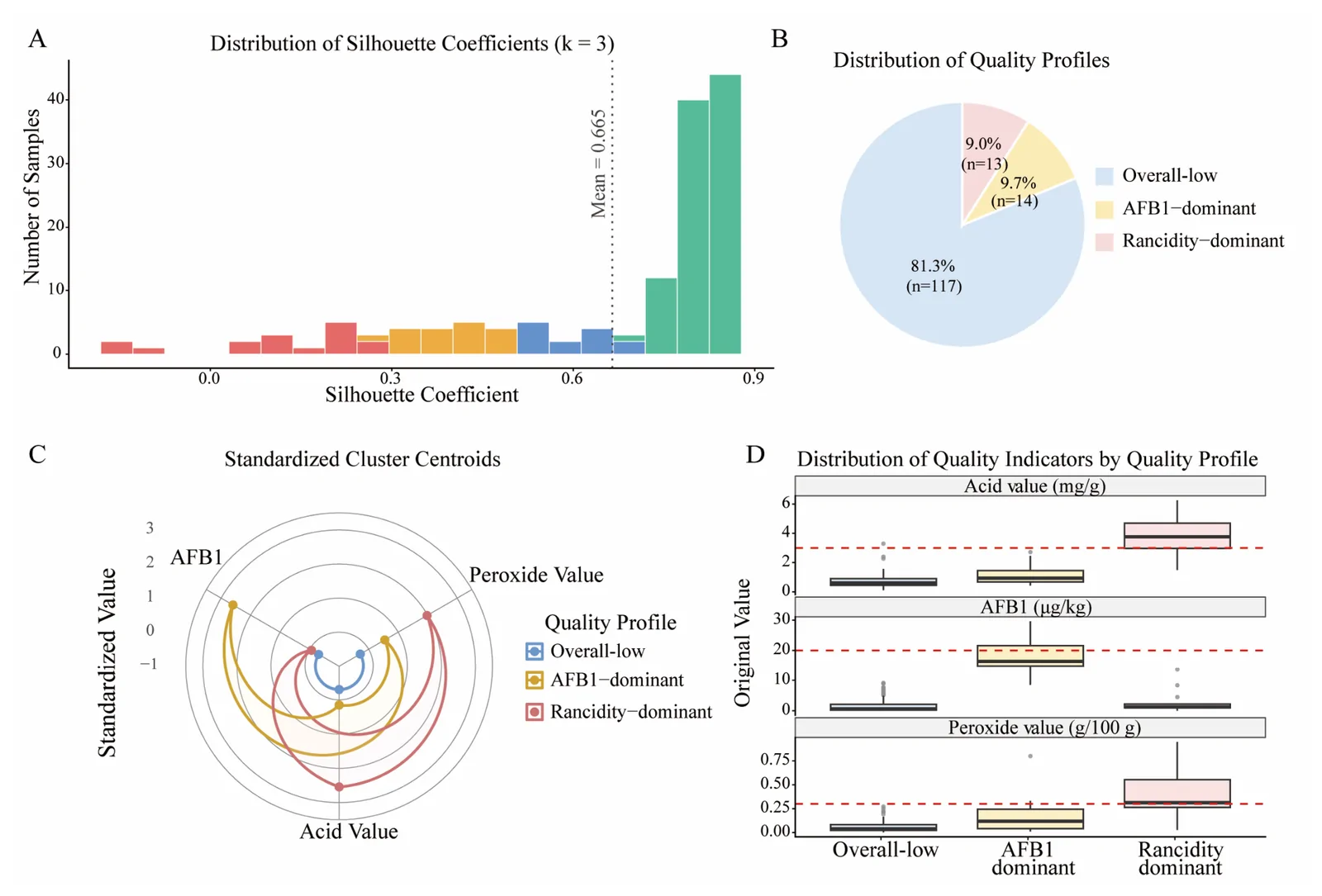
Quality profiles identified by K-means clustering. (**A**) Distribution of silhouette coefficients for k = 3; the dotted line indicates the mean value. (**B**) Distribution of samples across the three quality profiles. (**C**) Standardized cluster centroids. (**D**) Original-scale distributions of AFB1 concentration, acid value, and peroxide value across the quality profiles. Red dashed lines indicate the corresponding reference values.

The three-cluster solution yielded a mean silhouette coefficient of 0.6645 and a median of 0.8036, with only three samples showing negative silhouette values (Figure 3A). Most samples (*n* = 117, 81.2%) formed an overall-low profile, with comparatively low values across all three indicators. A second group (*n* = 14, 9.7%) showed the highest mean AFB1 concentration (17.9 μg/kg) and was designated the AFB1-dominant profile. The remaining 13 samples (9.0%) were characterized primarily by an elevated acid value (mean, 3.82 mg/g) and were designated the rancidity-dominant profile. These patterns were also evident in the standardized cluster centroids and the distributions of the original measurements (Figure 3B–D).

Sensitivity analysis after log_10_ transformation yielded a simpler two-cluster structure. Both the silhouette and Gap criteria favored k = 2, separating 74 samples (51.4%) with lower overall indicator levels from 70 samples (48.6%) with higher overall levels. All samples classified as AFB1-dominant (*n* = 14) or rancidity-dominant (*n* = 13) in the primary analysis were included in the higher-indicator group after transformation. When the transformed data were constrained to three clusters for direct comparison, 66.0% of samples retained their primary profile assignment (adjusted Rand index = 0.296) (Table S3).

Taken together, the analyses consistently identified a broad contrast between oils with lower and higher overall indicator levels, while the primary three-cluster analysis provided a finer description of samples characterized predominantly by elevated AFB1 or rancidity-related changes.

### 3.4. Dietary AFB1 Exposure and Modeled HCC Risk

**Figure 4:**
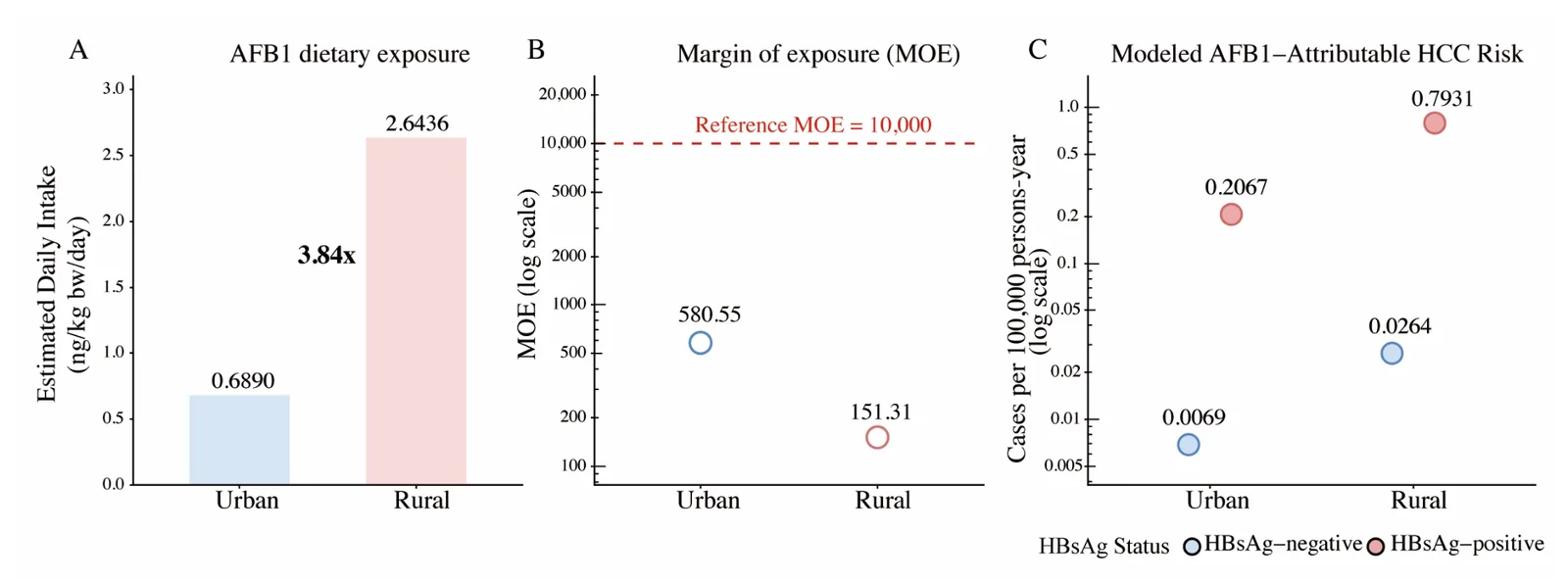
Dietary AFB1 exposure and modeled HCC risk under urban and rural scenarios. (**A**) Estimated daily intake (EDI); (**B**) margin of exposure (MOE), with the dashed line indicating the reference value of 10,000; and (**C**) modeled AFB1-attributable HCC risk according to HBsAg status. Panels (**B**,**C**) are shown on logarithmic scales. HCC, hepatocellular carcinoma; HBsAg, hepatitis B surface antigen.

The exposure assessment showed a clear contrast between the rural and urban scenarios. Estimated daily intake of AFB1 was 2.6436 ng/kg bw/day under the rural scenario, 3.84-fold higher than the 0.6890 ng/kg bw/day estimated under the urban scenario (Figure 4A). Correspondingly, the MOE was lower for the rural than for the urban scenario (151.31 vs. 580.55), with both values below the reference value of 10,000 (Figure 4B).

The same rural–urban contrast was observed for modeled AFB1-attributable HCC risk under both HBsAg scenarios. Under the HBsAg-negative scenario, the estimated risks were 0.0264 and 0.0069 cases per 100,000 person-years for the rural and urban scenarios, respectively. Under the HBsAg-positive scenario, the corresponding estimates were 0.7931 and 0.2067 cases per 100,000 person-years (Figure 4C). Overall, the rural scenario was characterized by higher estimated AFB1 exposure and modeled HCC risk, together with a lower MOE, than the urban scenario.

## 4. Discussion

This study provides a comprehensive assessment of AFB1 contamination, oil-quality characteristics, workshop hygiene, and modeled dietary risk in artisanal peanut oil produced by small-scale workshops in Nanning. AFB1 was frequently detected, although only a small proportion of samples exceeded the Chinese maximum level. Samples collected from counties consistently showed higher AFB1 concentrations, acid values, and peroxide values than those from municipal districts, indicating that regional differences extended beyond aflatoxin contamination to broader aspects of oil quality. The on-site assessment further showed that basic hygiene and spatial separation measures were more widely implemented than routine contaminant monitoring and aflatoxin-specific control practices. Together, these findings indicate substantial heterogeneity in the quality and safety of workshop-produced peanut oil and highlight the importance of considering both contaminant occurrence and deterioration-related quality indicators.

### 4.1. AFB1 Occurrence and Regional Differences in Peanut Oil Quality

The high frequency of detectable AFB1 in the present study is consistent with previous reports from southern China. In Guangdong Province, AFB1 was detected in 79.72% of 143 unpackaged peanut oil samples, with concentrations ranging from 0.02 to 174.13 μg/kg; 68 samples exceeded the Chinese maximum level of 20 μg/kg [15,16]. By comparison, AFB1 was detected in 88.2% of samples in the present study, whereas only 3.5% exceeded 20 μg/kg. Thus, contamination in the sampled workshops was characterized by frequent detection but relatively limited high-level exceedance. A nationwide analysis of peanuts and peanut oil also demonstrated marked geographic heterogeneity in aflatoxin contamination and exposure, with relatively high levels clustering in parts of South and East China [11].

The regional contrast observed in Nanning was also evident for acid and peroxide values, suggesting that the differences between counties and municipal districts were not restricted to AFB1 contamination. Several factors may contribute to such variation. Aflatoxin contamination can develop in peanut kernels before processing, and drought stress during cultivation has been associated with increased contamination risk [3,4]. Processing may further modify the amount of AFB1 transferred into peanut oil, with experimental studies showing substantial differences among extraction methods, including cold pressing and pressing after roasting [9]. These findings provide plausible context for the regional patterns observed here. However, because peanut origin, storage history, and detailed processing conditions were not systematically recorded, the county–municipal differences cannot be attributed to any single production or environmental factor.

### 4.2. Workshop Hygiene and Multivariate Quality Profiles

The on-site assessment provides additional context for the variation in peanut oil quality. Compliance was generally higher for basic environmental hygiene and spatial separation than for routine contaminant testing and aflatoxin-specific control measures, highlighting a gap between general workshop hygiene and contaminant-specific surveillance. Accordingly, visual screening cannot replace validated analytical testing, although rapid screening methods may complement confirmatory laboratory analysis [33,34]. This distinction is relevant because AFB1 contamination cannot be reliably identified through visual inspection alone [35]. Evidence from Guangzhou also supports the importance of production-level oversight: implementation of management regulations for small food workshops was associated with an immediate reduction and a sustained decline in AFB1 concentrations in home-made peanut oil [17]. These findings support the value of routine contaminant testing and process-level quality control alongside general hygiene requirements.

The simultaneous assessment of AFB1, acid value, and peroxide value provided a broader view of oil quality than aflatoxin testing alone [5]. Peroxide value is widely used as an indicator of primary lipid oxidation and therefore complements acid value in the assessment of edible-oil deterioration [36]. The primary clustering analysis identified a large overall-low profile and two smaller profiles characterized predominantly by elevated AFB1 or rancidity-related indicators. The mean AFB1 concentration in the AFB1-dominant profile was 17.9 μg/kg, approaching but remaining below the Chinese maximum level of 20 μg/kg. This profile therefore represents relatively elevated AFB1 rather than a group defined by regulatory non-compliance. By contrast, the rancidity-dominant profile was characterized mainly by deterioration-related indicators, showing that quality concerns may differ among oils even when AFB1 contamination is considered simultaneously.

The sensitivity analysis provided a complementary view of the clustering structure. After log_10_ transformation, a two-cluster solution was favored, but all samples assigned to the AFB1-dominant or rancidity-dominant profiles in the primary analysis remained within the broader higher-indicator group. Transformation therefore changed the preferred level of partitioning rather than eliminating the underlying contrast among samples. The two-cluster solution captured the broader separation in overall indicator levels, whereas the three-cluster solution provided additional information on whether the elevation was driven predominantly by AFB1 or by rancidity-related indicators. Accordingly, the three profiles are most appropriately interpreted as exploratory descriptive patterns within the sampled oils rather than as fixed classification categories.

### 4.3. Dietary Exposure and Modeled HCC Risk

The regional differences in AFB1 concentrations were reflected in the exposure assessment. The EDI under the rural scenario was 2.6436 ng/kg bw/day, compared with 0.6890 ng/kg bw/day under the urban scenario. Previous studies have reported substantial variation in AFB1 exposure associated with peanut oil. In Guangzhou, estimated daily AFB1 intake from home-made peanut oil ranged from 0.25 to 1.68 ng/kg bw/day between 2010 and 2022 [17]. At the national level, average aflatoxin exposure from peanuts and peanut oil was estimated at approximately 1.78–1.94 ng/kg bw/day, although regional estimates varied widely [11]. A prospective study in Guangxi reported a median estimated aflatoxin intake of 2.61 ng/kg bw/day from cooking oil among pregnant women, further supporting the local relevance of oil-borne exposure, although the study population and dietary-assessment methods differed from those used here [37]. These differences should not be interpreted as direct comparisons because exposure estimates depend on contamination distributions, dietary inputs, and study populations; nevertheless, they illustrate the strong influence of local contamination and consumption patterns on estimated dietary exposure.

Both MOE estimates in the present study were below 10,000, with a substantially lower value under the rural scenario. For genotoxic carcinogens such as AFB1, the MOE is used as a risk-characterization tool rather than as a direct measure of disease probability, and values below 10,000 are considered to indicate a potential public-health concern [1]. Modeled AFB1-attributable HCC risk showed the same rural–urban contrast and was higher under the HBsAg-positive scenario, consistent with the greater carcinogenic potency of AFB1 in the presence of chronic hepatitis B infection [1,32]. These estimates should therefore be interpreted as model-based scenarios derived from contamination data, population-level dietary parameters, and established potency factors rather than as observed HCC incidence. Overall, the exposure assessment reinforces the importance of reducing AFB1 contamination, particularly in workshop settings where higher concentrations were observed.

### 4.4. Limitations

Several limitations warrant consideration. Sampling was conducted during a single study period and therefore may not capture seasonal or longer-term variation in AFB1 contamination and oil quality. Exposure estimates were derived from regional mean AFB1 concentrations and population-level dietary parameters and should be interpreted as population-level scenarios rather than individual risk estimates. In addition, the absence of detailed information on peanut origin, storage history, and processing conditions limited further explanation of the observed regional differences.

## 5. Conclusions

AFB1 was frequently detected in artisanal peanut oil from small-scale workshops in Nanning, although exceedance of the regulatory limit was uncommon. Samples from counties consistently showed higher AFB1 concentrations, acid values, and peroxide values than those from municipal districts, indicating clear regional heterogeneity in both contamination and oil quality. The on-site assessment further showed that routine contaminant testing and aflatoxin-specific control were less frequently implemented than basic hygiene measures. Multivariate analysis identified additional heterogeneity in quality profiles, while the exposure assessment showed higher estimated AFB1 exposure, lower MOE, and higher modeled HCC risk under the rural scenario. Taken together, these findings highlight the importance of integrating routine AFB1 monitoring with broader quality and storage management in small-scale peanut-oil production, with particular attention to workshops in county settings.

## Figures and Tables

**Table 1:** Hygiene and food-safety compliance among small-scale peanut-oil workshops (*n* = 144).

Domain	Assessment Item	Compliance, %
Production environment hygiene	Adequate ventilation without foul or offensive odors	87%
	Floors and walls free from visible contamination	61%
	No garbage accumulation	78%
	Appropriate temperature and moisture conditions during storage	78%
	Equipment clean and free of oil residues	60%
Personnel and raw material management	Separation of poultry/pet areas from production areas	84%
	Separation of production and living areas	84%
	Production personnel wearing clean work clothing	53%
	Production qualification documents prominently displayed	68%
	Raw materials and equipment maintained > 2 m from potential contamination sources (e.g., sewage)	88%
Aflatoxin prevention and control	Routine testing for contaminants	18%
	Availability of aflatoxin-degradation equipment	35%
	Intention to purchase aflatoxin-degradation equipment	62%

## Data Availability

The raw data underlying the analyses presented in this study are provided in Supplementary Table S4.

## Supplementary Materials

The following supporting information can be downloaded at: https://www.mdpi.com/article/10.3390/foods15193493/s1, Table S1: Structured on-site hygiene and food-safety assessment for small-scale peanut-oil workshops; Table S2: Regional descriptive statistics, normality assessment, and Mann—Whitney comparisons of peanut-oil quality indicators; Table S3: Detailed characteristics, standardized centers, silhouette diagnostics, and sensitivity analyses of K-means quality profiles (*n* = 144); Table S4. Sample-level dataset used for the analysis of workshop hygiene and peanut-oil quality indicators (*n* = 144).
